# Is p53 Immunohistochemistry a Reliable Indicator of *TP53* mutation? An NGS-based assessment in lung adenocarcinoma

**DOI:** 10.12669/pjms.42.5.14704

**Published:** 2026-05

**Authors:** Gizem Teoman, Zeynep Sagnak Yilmaz, Safak Ersoz, Sevdegul Aydin Mungan

**Affiliations:** 1 Gizem Teoman, MD Assistant Professor, Department of Medical Pathology, Karadeniz Technical University, Faculty of Medicine, Trabzon, Turkey; 2 Zeynep Sagnak Yilmaz, MD Assistant Professor, Department of Medical Pathology, Karadeniz Technical University, Faculty of Medicine, Trabzon, Turkey; 3 Safak Ersoz, MD Professor, Department of Medical Pathology, Karadeniz Technical University, Faculty of Medicine, Trabzon, Turkey; 4 Sevdegul Aydin Mungan, MD Professor, Department of Medical Pathology, Karadeniz Technical University, Faculty of Medicine, Trabzon, Turkey

**Keywords:** Lung adenocarcinoma, Missense mutation, Next-generation sequencing, p53 immunohistochemistry, Surrogate biomarker, *TP53*

## Abstract

**Objective:**

To evaluate the concordance between p53 immunohistochemistry (IHC) patterns and *TP53* mutation types identified by next-generation sequencing (NGS) in lung adenocarcinoma.

**Methodology:**

In this retrospective study, 166 lung adenocarcinoma cases diagnosed between 2020 and 2025 at the Department of Medical Pathology, Karadeniz Technical University Faculty of Medicine were analyzed. p53 IHC staining was classified as overexpression, null, or wild-type. *TP53* mutations detected by NGS were categorized as missense, nonsense, frameshift, or splice-site variants. Sensitivity, specificity, positive predictive value (PPV), and negative predictive value (NPV) of each IHC pattern for predicting its corresponding mutation class were calculated using NGS as the reference standard.

**Results:**

p53 IHC showed overexpression in one hundred eighteen cases (71.1%), a null pattern in forty-two (25.3%), and wild-type staining in six (3.6%). *TP53* mutations included missense (74.1%), nonsense (11.4%), frameshift (10.2%), and splice-site variants (4.3%). Overexpression strongly correlated with missense mutations: 117 of 118 overexpression cases (99.2%) carried missense variants. The null pattern was mainly associated with truncating mutations, including nonsense and frameshift variants. Wild-type staining showed heterogeneous profiles. Overexpression predicted missense mutations with a sensitivity of 95.1%, specificity of 97.7%, PPV of 99.2%, and NPV of 87.5%. The null pattern predicted nonsense mutations with moderate PPV but high NPV.

**Conclusions:**

p53 IHC is a reliable surrogate marker for identifying TP53 missense mutations in lung adenocarcinoma, with overexpression demonstrating excellent diagnostic accuracy. Although the null pattern has limited PPV for nonsense mutations, its high NPV supports its use in excluding such variants. p53 IHC may serve as a practical and cost-effective approach for inferring TP53 mutation status, particularly in settings where molecular testing is restricted.

## INTRODUCTION

Lung adenocarcinoma, the most common subtype of non-small cell lung carcinoma (NSCLC), remains a major cause of cancer-related mortality worldwide.^1^ Molecular alterations play a key role in its behavior and therapeutic response, and among these, *TP53* is one of the most frequently mutated genes. The p53 protein, encoded by *TP53*, regulates DNA repair, cell cycle control, senescence, and apoptosis. Mutations in *TP53* disrupt these pathways, promoting genomic instability and tumor progression.^2^

Immunohistochemistry (IHC) for p53 is widely used in routine pathology as a practical surrogate marker for *TP53* mutation status. Three characteristic staining patterns have been described: (1) Overexpression, typically associated with missense mutations; (2) Null pattern, corresponding to truncating mutations such as nonsense or frameshift variants; (3) Wild-type pattern, reflecting normal p53 turnover.^3^

Although next-generation sequencing (NGS) is the gold standard for detecting *TP53* mutations, its use may be limited by cost, technical requirements, and accessibility. In contrast, p53 IHC is rapid, inexpensive, and readily applicable to formalin-fixed paraffin-embedded tissue. Establishing a reliable correlation between p53 IHC patterns and underlying *TP53* mutation types therefore holds significant diagnostic and practical value.^4^

Previous studies in various tumor types have shown strong concordance between p53 IHC patterns and *TP53* mutation classes; however, evidence in lung adenocarcinoma remains limited and variable. Differences in methodology, interpretation criteria, and tumor heterogeneity have contributed to inconsistent results.^5^

The aim of the present study was to evaluate the concordance between p53 IHC expression patterns and *TP53* mutation types detected by NGS in lung adenocarcinoma. Demonstrating reliable associations may support the use of p53 IHC as a cost-effective surrogate tool in diagnostic workflows, particularly in settings where molecular testing is not routinely available.

## METHODOLOGY

This retrospective study included 166 lung adenocarcinoma cases diagnosed between January 2020 to September 2025 in the archives of the Department of Medical Pathology, Karadeniz Technical University Faculty of Medicine. All cases had undergone next-generation sequencing (NGS) analysis with documented *TP53* mutation status. Only cases with a confirmed histopathological diagnosis and available formalin-fixed, paraffin-embedded (FFPE) tissue blocks suitable for additional immunohistochemical evaluation were included.

Cases submitted as external consultations, or those for which archival tissue blocks/slides were unavailable, were excluded from the study. Since both NGS results and tissue blocks for immunohistochemistry were already present in the institutional archives, no additional tissue sampling or study-specific funding was required.

This study was approved by the Karadeniz Technical University Faculty of Medicine Scientific Research Ethics Committee (Approval No: 2025/262; Date: October 07, 2025).

### Immunohistochemical Evaluation of p53:

p53 immunohistochemistry was performed retrospectively on FFPE tumor sections using standardized automated staining protocols (Ventana BenchMark ULTRA, Roche Diagnostics, Tucson, AZ, USA). Nuclear immunoreactivity was evaluated and classified into three patterns:

- ***Overexpression pattern:*** strong, diffuse nuclear staining in >80% of tumor cells.
- ***Null pattern:*** complete absence of tumor cell nuclear staining with preserved internal controls (stromal/inflammatory cells).
- ***Wild-type pattern:*** variable, weak-to-moderate nuclear staining in a subset of tumor cells.

All slides were independently reviewed by two experienced pathologists, blinded to NGS results, to ensure consistency in the interpretation of staining patterns.

### DNA extraction and TP53 mutation assay:

Somatic *TP53* mutations were analyzed using NGS. Depending on the diagnostic period, one of the following DNA-based NGS panels was used: QIAseq Solid Custom MSI Panel, QIAact AIT DNA UMI Panel, or the QIAact Actionable Insights Tumor (AIT) Panel. DNA extraction was performed from FFPE tumor tissue using the *QIAGEN GeneRead DNA/RNA FFPE Kit* (QIAGEN, Hilden, Germany). DNA concentration was measured with the Qubit 4 Fluorometer, and DNA quality was assessed via Qiaxcel electrophoresis. Sequencing libraries were prepared according to the manufacturers’ protocols and sequenced on the Illumina NovaSeq platform. Bioinformatic analysis was conducted using Qiagen Clinical Insight (QCI) Interpret and CLC Genomic Workbench. For FFPE-derived DNA, variants were included if they met the following quality thresholds: a minimum read depth ≥500×, allele frequency ≥5%, and quality score (QUAL) >200. Variants were classified according to international tiered classification guidelines, and only Tier I–II (pathogenic/likely pathogenic) variants were included in the final analysis. Detected *TP53* variants were further categorized based on molecular type and predicted functional impact: missense mutations were evaluated according to their position within the DNA-binding domain; nonsense and frameshift variants were interpreted as loss-of-function alterations; and splice-site mutations were considered disruptive to mRNA processing. All variants were cross-validated using population databases, COSMIC, and functional prediction tools.

### Statistical analysis:

Immunohistochemical and molecular data were extracted from pathology reports and entered into a dedicated SPSS database. The distribution of p53 immunohistochemical patterns (wild-type, overexpression, null) and *TP53* mutation types (missense, nonsense, frameshift, splice-site) was summarized using descriptive statistics. The concordance between each p53 IHC pattern and the corresponding *TP53* mutation spectrum was evaluated. For the purpose of assessing predictive performance, the sensitivity, specificity, positive predictive value (PPV), and negative predictive value (NPV) of p53 IHC in predicting the presence of *TP53* mutations (and specifically missense mutations) were calculated using NGS as the reference standard. All statistical analyses were conducted using SPSS software (IBM SPSS Statistics, version 27.0; IBM Corp., Armonk, NY, USA).

## RESULTS

A total of 166 lung adenocarcinoma cases were included in the study. Analysis of p53 immunohistochemistry (IHC) revealed 118 cases (71.1%) with overexpression, 42 cases (25.3%) with a null pattern, and 6 cases (3.6%) with wild-type staining. Regarding *TP53* mutational status, 123 cases (74.1%) harbored missense mutations, followed by 19 cases (11.4%) nonsense, 17 cases (10.2%) frameshift, and seven cases (4.3%) splice-site mutations. Evaluation of concordance between p53 IHC patterns and *TP53* mutation types demonstrated strong associations. Among the 118 cases with p53 overexpression, 117 (99.2%) harbored missense mutations, while one case showed a splice-site mutation. In the 42 null-pattern cases, 17 (40.5%) carried nonsense mutations, 16 (38.1%) frameshift, 5 (11.9%) splice-site, and 4 (9.5%) missense mutations. Among the 6 wild-type IHC cases, mutations included two missense (33.3%), two nonsense (33.3%), one frameshift (16.7%), and one splice-site (16.7%). The immunohistochemical staining patterns and corresponding *TP53* mutational profiles of all cases are summarized in Table-I.

**Table-I:** Immunohistochemical staining patterns and mutational analysis of TP53.

Mutation type		Immunohistochemical pattern of p53 (n)		Total (n)
	Overexpression	Null	Wild	
Missense	117	4	2	123
Nonsense	0	17	2	19
Frameshift	0	16	1	17
Splice-site	1	5	1	7

**Fig.1:**
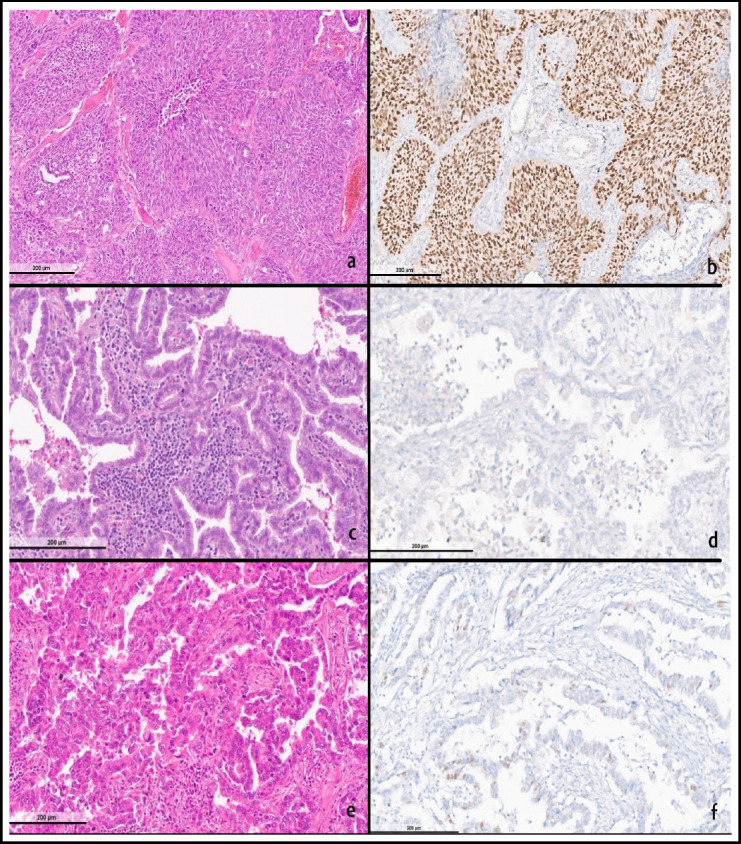
Representative histopathological and immunohistochemical features of *TP53* mutation–associated p53 staining patterns in lung adenocarcinoma. (a) Hematoxylin–eosin (H&E) stained section of a lung adenocarcinoma harboring a missense *TP53* mutation. (b) Corresponding p53 immunohistochemistry showing strong diffuse nuclear overexpression, characteristic of missense mutations. (c) H&E section of a lung adenocarcinoma with a nonsense *TP53* mutation. (d) p53 immunohistochemistry demonstrating a complete absence of nuclear staining (null pattern), consistent with nonsense/frameshift mutations. (e) H&E section from a tumor carrying a splice-site *TP53* mutation. (f) p53 immunohistochemistry displaying a wild-type staining pattern, with heterogeneous and weak nuclear positivity.

Fig.1 illustrates representative histopathological and immunohistochemical features of lung adenocarcinomas with different *TP53* mutation types, demonstrating missense mutations with diffuse p53 overexpression, nonsense/frameshift mutations with complete absence of staining (null pattern), and splice-site mutations exhibiting a wild-type heterogeneous staining pattern.

Diagnostic performance analysis showed that p53 overexpression predicted missense mutations with a sensitivity of 95.1%, specificity of 97.7%, positive predictive value (PPV) of 117/118 (99.2%), and negative predictive value (NPV) of 42/48 (87.5%). Similarly, the p53 null pattern predicted nonsense mutations with a sensitivity of 89.5%, specificity of 83.0%, PPV of 17/42 (40.5%), and NPV of 122/124 (98.4%).

These findings demonstrate that p53 IHC, particularly the overexpression pattern, is a highly reliable surrogate marker for identifying *TP53* missense mutations in lung adenocarcinoma, whereas the null pattern shows moderate predictive value for nonsense mutations but retains excellent NPV.

## DISCUSSION

In this retrospective cohort of lung adenocarcinoma, we demonstrated a strong concordance between p53 IHC patterns and *TP53* mutational categories defined by NGS. The overexpression pattern was almost exclusively associated with missense mutations (99.2%), whereas the null pattern was predominantly linked to truncating alterations (nonsense, frameshift, splice-site). These findings support the biologic rationale that missense mutations stabilize mutant p53 protein leading to nuclear accumulation, while truncating mutations result in loss of detectable protein expression.^4^,^5^

*TP53* is among the most frequently mutated genes in lung adenocarcinoma, reported in approximately 45–60% of cases depending on cohort composition and sequencing platform.^2^ In The Cancer Genome Atlas (TCGA) and subsequent large genomic studies, the majority of *TP53* mutations in lung adenocarcinoma are missense alterations located in the DNA-binding domain, with a smaller fraction comprising nonsense and frameshift variants.^6^ Our mutation distribution (74.1% missense) is concordant with these molecular epidemiologic data, reinforcing the representativeness of our cohort.

Several recent investigations have examined the predictive value of p53 IHC for *TP53* mutation status in solid tumors. Sung et al. reported that p53 IHC “overexpression” reliably predicted in-frame and DNA-binding domain mutations, while the “null” pattern strongly correlated with truncating mutations.^4^ Similarly, a 2025 NSCLC-based study demonstrated that pattern-based interpretation achieved 92% concordance with NGS-defined *TP53* alterations.^5^

In lung adenocarcinoma specifically, Kurihara et al. showed that p53 IHC status closely paralleled *TP53* mutation and was associated with early relapse after surgical resection.^7^ Our findings further strengthen this evidence by providing high predictive values: overexpression predicted missense mutations with PPV 99.2% and specificity 97.7%. This level of diagnostic performance is comparable to or exceeds previously reported sensitivities (86–95%) in other tumor types.^4^,^8^

Notably, pattern-based interpretation has gained increasing acceptance across tumor systems, including colorectal, endometrial, and gynecologic malignancies.^8^-^10^ The consistency of these results across organ systems suggests that the biologic consequences of *TP53* mutations on protein stability are conserved, making p53 IHC a broadly applicable surrogate tool.

### Implications for clinical practice:

From a practical standpoint, our findings have direct implications for routine diagnostic pathology. In settings where comprehensive NGS is not immediately available or cost-effective, p53 immunohistochemistry may serve as an effective initial triage tool. Strong diffuse nuclear overexpression strongly suggests an underlying missense mutation, whereas a complete absence of staining (null pattern) raises suspicion for truncating alterations, reflecting the established impact of *TP53* mutation type on protein stability. This approach may be particularly relevant in resource-limited environments, where rapid molecular stratification remains challenging. Notably, recent NGS-based data from Pakistan have demonstrated that *TP53* mutations constitute a substantial proportion of pathogenic genomic alterations in solid tumors, underscoring the clinical importance of accessible surrogate markers in such settings.^11^

Additionally, given emerging evidence that *TP53* mutation subtype may influence tumor biology, immune microenvironment, and therapeutic resistance patterns, rapid identification of mutation category may assist in risk stratification.^12^-^14^

Recent studies in lung adenocarcinoma have also explored the relationship between *TP53* alterations and immune-related markers, genomic instability, and treatment outcomes.^15^ As precision oncology evolves, integrating morphologic, immunohistochemical, and molecular data will become increasingly important.^16^ Our results suggest that p53 IHC can serve as a cost-effective bridge between morphology and genomics.

### Discordant cases and Biological considerations:

Despite high concordance, a small subset of discordant cases was observed. One overexpression-pattern tumor harbored a splice-site mutation, and several null-pattern or wild-type IHC cases carried non-truncating mutations. Such discrepancies have been previously reported.^17^

### Several mechanisms may explain discordance:

- Not all missense mutations produce stable proteins detectable by IHC.^18^
- Certain splice-site alterations may generate partially stable truncated proteins.^19^
- Post-translational regulation of p53 (e.g., MDM2-mediated degradation) may reduce detectable protein levels independent of mutation class.^20^
- Technical factors such as fixation quality and antibody clone variability can also influence staining interpretation.^21^
- Therefore, while highly predictive, p53 IHC should not fully replace molecular testing in clinically critical scenarios.

### Strengths of the study:

Our study possesses several important strengths that enhance the robustness and clinical relevance of our findings. First, *TP53* mutational status was determined using next-generation sequencing (NGS), which is considered the gold standard for molecular characterization. Second, the inclusion of a relatively large, well-defined single-institution cohort of lung adenocarcinoma cases increases internal consistency in pre-analytical and analytical procedures. Third, strict quality control thresholds for variant inclusion (read depth, allele frequency, and quality score) minimized the risk of false-positive or low-confidence mutations.

In addition, mutations were carefully subclassified according to molecular type and predicted functional impact, allowing a more nuanced correlation between genetic alterations and immunohistochemical patterns. Finally, all p53 IHC slides were independently evaluated by two experienced pathologists blinded to the NGS results, reducing observer bias and strengthening the validity of the concordance analysis. Collectively, these methodological features enhance the reliability and reproducibility of our results.

### Recommendations:

Based on our findings, we recommend:

- Utilization of p53 IHC as an initial screening tool in diagnostic practice for lung adenocarcinoma, especially where NGS is not immediately available. Overexpression (strong diffuse nuclear positivity) should prompt consideration of *TP53* missense mutation, while null pattern should raise suspicion of truncating mutations.
- Performing confirmatory NGS in clinically relevant scenarios, especially if IHC and clinical/therapeutic context are discordant — for instance, null-pattern tumors with high-grade or aggressive behavior, or wild-type IHC tumors with clinical features suggestive of *TP53* mutation.
- Standardization of IHC protocols and interpretation criteria, including defined cut-offs and dual-reader review, to maximize reproducibility and minimize false-negative/positive rates.
- While our study demonstrates a strong concordance between p53 IHC patterns and *TP53* mutation categories, further investigations are warranted. Prospective multicenter studies could validate these findings across diverse populations and sequencing platforms. In addition, the clinical impact of discordant cases, the potential influence of *TP53* mutation subtypes on treatment response, tumor immune microenvironment, and patient outcomes require further exploration. Finally, integrating p53 IHC with emerging molecular and genomic biomarkers may improve risk stratification and guide personalized therapy in lung adenocarcinoma.

### Limitations:

Despite the strong overall concordance, some limitations must be acknowledged. First, a minority of cases showed discordance: e.g., a single overexpression-pattern case with splice-site mutation, and null- or wild-type-pattern cases harboring non-truncating mutations. Such discrepancies may arise from several factors:

- Functional consequences of different mutation types: not all *TP53* mutations, even within the same class, result in similar degradation/stability of p53 protein, affecting IHC detectability. Indeed, prior studies reported reduced sensitivity of p53 IHC for some loss-of-function mutations, particularly splice–site or rare nonsense variants.^22^
- Influence of non-genetic regulation of p53 (e.g. MDM2 amplification, degradation pathways), especially in truncated p53, potentially leading to absent staining despite wild-type or non-truncating mutations.^23^

## CONCLUSION

Our study demonstrates that p53 immunohistochemistry — when interpreted with a pattern-based approach — shows high concordance with *TP53* mutational status in lung adenocarcinoma, particularly for missense mutations. While not infallible, p53 IHC represents a cost-effective, rapid surrogate marker and may be integrated into routine diagnostic workflow, with NGS reserved for confirmation in ambiguous or clinically significant cases.

### Author’s Contribution:

**GT:** Conceived, designed and did statistical analysis & editing of manuscript, is responsible for integrity of research.

**GT, ZSY and SE:** Did data collection and manuscript writing.

**GT and SAM:** Did review and final approval of manuscript.
